# The "Snacking Child" and its social network: some insights from an italian survey

**DOI:** 10.1186/1475-2891-10-132

**Published:** 2011-11-29

**Authors:** Dario Gregori, Francesca Foltran, Marco Ghidina, Federica Zobec, Simonetta Ballali, Laura Franchin, Paola Berchialla

**Affiliations:** 1 Laboratory of Epidemiological Methods and Biostatistics, Department of Environmental Medicine and Public Health, University of Padova, via Loredan 18, 35031 Padova, Italy; 2 ZETA Research Inc., via Caccia 8, 34129 Trieste, Italy; 3 Prochild ONLUS, Piazza San Giovanni 2, 34122 Trieste, Italy; 4 Department of Public Health and Microbiology, University of Torino, via Santena 5 bis, 10126 Torino, Italy

**Keywords:** snacks, overweight in children, social networks, physical activity

## Abstract

**Background:**

The hypothesis underlying this work is that the social network of a child might have an impact on the alimentary behaviors, in particular for what concerns snack consumption patterns.

**Methods:**

1215 Italian children 6-10 ys old were interviewed using a CATI facility in January 2010. 608 "snackers" and 607 "no-snackers" were identified. Information regarding family composition, child and relatives BMI, mother perception of child weight, child, father and mother physical activity, TV watching, social network, leisure time habits and dietary habits of peers, were collected. Association of variables with the status of snacker was investigated using a multivariable logistic regression model.

**Results:**

Snackers children seem to be part of more numerous social network (1.40 friends vs 1.14, p = 0.042) where the majority of peers are also eating snacks, this percentage being significantly higher (89.5 vs 76.3, p < 0.001) than in the "no-snacker" group. The snacking group is identified by the fact that it tends to practice at least 4 hours per week of physical activity (OR: 1.36, CI: 1.03-1.9). No evidence of an association between snacking consumption and overweight status has been shown by our study.

**Conclusions:**

The snacking child has more active peer-to-peer social relationships, mostly related with sport activities. However, spending leisure time in sportive activities implies being part of a social environment which is definitely a positive one from the point of view of obesity control, and indeed, no increase of overweight/obesity is seen in relation to snack consumption.

## Background

Child obesity has been described as the product of an interaction between a susceptible host and an environment promoting the disease, and results when energy intake exceeds energy expenditure [1,2]. Studies finalized to identify behaviors acting as obesity promoters have been mainly focused on evaluating the role of dietary habits in terms of energy intake source [3], and the role of physical activity as a way to increase the energy expenditure [4]. Moreover, recent studies have been addressed to understand individual, familiar and environmental factors determining behaviors suspected to be unhealthy [5,6].

Among alimentary behaviors, snacking activity has been identified as one potential cause of obesity and/or overweight in childhood and adolescent [7]. Following Hampl [8], a child who's getting more than 15-20% of his/her daily caloric intake from snacks can be defined as a "snacker". Snacks are suspected to be obesity promoters because they are high-fat and high-sugar foods. Nevertheless, even if the percentage of energy from dietary fat or from sugar has been widely investigated as determinant of body fat accumulation, the existence of causal relationships between single nutrients or foods and obesity is controversial. In fact, while cross-sectional studies seem to be suggestive of an association, prospective cohort studies have frequently failed in finding such a correlation. Moreover, snacking has been related with other behaviors, such as TV watching, which are all believed to be, by itself, obesogenic; in this way, a pattern of potentially unhealthy behaviors has been described, identifying snacking children as having a sedentary lifestyle and spending the majority of leisure time alone [9]. However, snacking is also related with social and group activities, as an accompanying activity during social meetings and after them [10]. Social pattern of interaction are of course very different when referred to adults than for children. Nevertheless, for both, the relevant support provided by social networks in maintaining health and well-being has been recently recognized, but the joint effect of snacking activity and social relationships on obesity has been not really investigated, although several reports suggest the existence of an inverse relation among these factors [11-13].

The hypothesis underlying this work is that the social network might have an impact on the alimentary behaviors of the child, and in particular, for what is concerning the snacking activity, that: (i) "snacking" is not an individual or family-restricted choice, but instead that the involvement of peers is relevant with respect to this behavior, thus implying that the probability of being a "snacker" is higher in a context were other peers are also "snackers", and that (ii) increased calories intake due to "snacking" might be even compensated by a child who is inserted in a social network, if the latter is active from the perspective of exercise and physical activity.

To this purposes, a survey on children in Italy has been conducted, identifying both a group of "snackers" and one of "no-snackers", and comparing them in terms of social networks and leisure time behaviors, as well as in terms of overweight prevalence.

## Materials and methods

### Study conduction and identification of the social network

The survey has been conducted using a CATI (Computer Assisted Telephone Interview) facility on 1215 children age 6-10 years, in January 2010 in Italy. Calls have been conducted by experienced CATI operator. The sample has been stratified according to the definition of snacker, thus collecting information on 608 "snackers" and 607 "no-snackers" children.

One-thousand two-hundred fifteen mothers, randomly selected through an automated system afferent to the Italian landline phone directory have been asked to answer directly on their child and on the group of the closest friends of him/her, defined within a limit of 10 children per referenced child. The random sample was constructed in a way to approximately provide an equal geographical distribution of the families in the North, in the Center and in the South of Italy.

### Interviews

The study is based on telephone interview on a randomly chosen set of mothers living in Italy and with a cable-phone connection. Informed consent was obtained from people interviewed before starting the set of questions.

First, mothers answering to the phone calls were asked to provide information useful for stratifying the child as "snacker" or "no-snacker". Thus, the questionnaire was divided into 4 parts. The first part investigated on basic sociodemographic characteristics, like education, composition of the family and work. In the second section mothers were asked to answer about family's physical characteristics, like weight and height. The third part assessed the behavioral aspects of their children, like weekly hours of physical activity and more details on the consumption patterns of snacks. The latter part regarded children's social network; mothers were asked to answer on their children's friends, up to a maximum of 10 children, on snacking habits, physical activities and perception of their nutritional status.

### Definitions

The "snacker" has been defined as consuming at least 3 or more of the following products per day: filled cakes/sweet snacks, plain cakes/sweet snacks, chocolate snacks, sodas (no sugar-free) in can or bottle. This adds up to an average of at least 360-400 calories, which counts up to 18-22% of the recommended daily caloric intake for a child (about 1700-1900 calories per day in the age class 6-10) and it is coherent with the definition of the "snacker" as provided in the literature [8].

Body mass index (BMI) was calculated as weight (kg)/height^2 ^(m). According to the present guidelines, in adults, normal weight, overweight and obesity were defined as a BMI < 25.0 kg/m^2^, 25.0- 29.9 kg/m^2^, and ≥ 30.0 kg/m^2^, respectively [14]. Data on weight and height of the child, used to compute the BMI were updated in 76% of the cases to the last three months and they, in the remaining 24%, never exceeded the year. Children were defined as obese according to the percentile to which they were belonging, according to their age, as indicated from the CDC (Centers for Disease Control and Prevention) pediatric growths charts [15]; thus, they were defined as "at risk for overweight" if their weight was between the 85^th ^and the 95^th ^percentile and as "overweight" is greater than the 95^th ^percentile [16]. CDC pediatrics growth charts were chosen as recommended when assessing growth's parameters of children older than 2 years [17,18].

### Statistical Methods

Continuous variables are expressed as median and inter-quartile difference and categorical variables as percentages and absolute numbers. Differences between groups were compared using Wilcoxon and McNemar or Chi-Square tests [19,20], as appropriate.

All variables investigated are listed in Tables 1 and 2. Association of variables with the status of snacker has been investigated using a multivariable logistic regression model. Association of variables with the BMI level of the children in the "snacker" group has been investigated using a multivariable linear regression model. The following model building strategy applies to both models. Variables related (with a maximum p-value of the association, as resulting in the univariable analysis, of 0.25), to the network around the snacker have been also inserted in the model and final estimates have been adjusted for inter-network correlation using a Huber-White estimating equation approach.

**Table 1:** Description of the families surveyed.

	***N***	***Snacker***	***No snacker***	***Overall***	***p-value^1^***	***BMI***	***p-value^2^***
		***608***	***607***	***1215***		**(Snackers only)**	
	
**Number of children in the family**					p < 0.001		p = 0.232
One		63 (10.4)	109 (18.0)	172 (14.2)		17.6 [15.2; 19.2]	
Two or more		545 (89.6)	498 (82.0)	1043 (85.8)		17.3 [15.3; 20.4]	
**Number of Children in the family aged 6-10 yrs**					p = 0.001		p = 0.047
One		395 (65.0)	450 (74.1)	845 (69.5)		17.8 [15.3; 20.4]	
Two or more		213 (35.0)	157 (25.9)	370 (30.5)		16.6 [15.3; 19.0]	
**Family Composition**					p = 0.003		p = 0.017
Normal (father, mother and children)		527 (86.7)	558 (91.9)	1085 (89.3)		17.6 [15.3; 19.2]	
Enlarged (normal with grandparents or other relatives living together)		81 (13.3)	49 (8.1)	130 (10.7)		16.6 [16.0; 23.1]	
**Father's Education**	1213				p < 0.001		p = 0.005
Up to the mandatory school		312 (51.3)	233 (38.5)	545 (44.9)		17.6 [15.9; 20.4]	
High school degree		225 (37.0)	270 (44.6)	495 (40.8)		17.8 [15.2; 19.2]	
BS or more		71 (11.7)	102 (16.9)	173 (14.3)		15.9 [13.9; 20.0]	
**Mother's Education**	1208				p = 0.001		p = 0.060
Up to the mandatory school		246 (40.8)	208 (34.4)	454 (37.6)		17.8 [16.0; 20.7]	
High school degree		306 (50.7)	309 (51.1)	615 (50.9)		17.3 [15.2; 19.0]	
BS or more		51 (8.5)	88 (14.5)	139 (11.5)		17.5 [14.5; 19.4]	
**Father's Job**	1211				p = ns		p = 0.005
Independent worker		221 (36.3)	237 (39.3)	458 (37.8)		18.1 [15.3; 20.4]	
Dependent worker		377 (62.0)	359 (59.5)	736 (60.8)		17.1 [15.3; 19.2]	
Other (retired, unemployed)		10 (1.6)	7 (1.2)	17 (1.4)		15.3 [15.3; 15.3]	
**Mother's Job**	1207				p = ns		p = 0.010
Independent worker		91 (15.1)	89 (14.7)	180 (14.9)		16.6 [15.9; 18.2]	
Dependent worker		247 (41.0)	257 (42.5)	504 (41.8)		17.3 [14.9; 19.0]	
Other (retired, unemployed, housewife)		265 (43.9)	258 (42.7)	523 (43.3)		17.8 [15.9; 20.8]	
**Mother has a full time job (40 hrs per week)**	1207				p = ns		p = 0.053
Yes		129 (38.7)	134 (39.0)	263 (38.8)		16.5 [14.9; 19.0]	
No		204 (61.3)	210 (61.0)	414 (61.2)		17.8 [15.3; 18.3]	
**Who is taking care of the child outside schooltime**	1213				p = 0.009		p < 0.001
Mother/Father		499 (82.1)	529 (87.4)	1028 (84.7)		17.6 [15.9; 20.4]	
Other people		109 (17.9)	76 (12.6)	185 (15.3)		15.3 [14.6; 18.8]	
**Exercise Time (hours per week) of the mother**	1175				p < 0.001		p < 0.001
None		428 (74.8)	416 (69.0)	844 (71.8)		16.7 [15.3; 18.8]	
Between one and three hours		68 (11.9)	138 (22.9)	206 (17.5)		19.0 [17.8; 20.8]	
Four hours or more		76 (13.3)	49 (8.1)	125 (10.6)		18.3 [14.9; 20.4]	
**Mother's BMI**	1161	23 [21; 24]	22 [20; 24]	22 [20; 24]	p = 0.015		p < 0.001
Underweight/normal		454 (79.9)	472 (79.6)	926 (79.8)	p = 0.002	16.7 [15.3; 18.8]	
Overweight		72 (12.7)	101 (17.0)	173 (14.9)		20.0 [17.6; 20.4]	
Obese		42 (7.4)	20 (3.4)	62 (5.3)		21.4 [14.3; 23.4]	
**Exercise Time (hours per week) of the father**	1176				p = ns		p = 0.025
None		425 (73.7)	424 (70.8)	849 (72.2)		17.4 [15.8; 19.2]	
Between one and three hours		93 (16.1)	100 (16.7)	193 (16.4)		17.8 [15.3; 20.8]	
Between four and six hours		43 (7.5)	57 (9.5)	100 (8.5)		18.3 [14.9; 19.2]	
Seven hours or more		16 (2.8)	18 (3.0)	34 (2.9)		14.8 [11.6; 22.4]	
**Father's BMI**	1100	25 [24; 27]	25 [24; 27]	25 [24; 27]	p = 0.152		p < 0.001
Underweight/normal		265 (48.4)	285 (51.5)	550 (50.0)	p = ns	16.6 [15.3; 18.7]	
Overweight		243 (44.4)	227 (41.0)	470 (42.7)		17.8 [15.1; 20.7]	
Obese		39 (7.1)	41 (7.4)	80 (7.3)		18.3 [17.6; 22.8]	
**Nationality of the family**	1193				p = ns		p = 0.003
Italian		557 (94.7)	579 (95.7)	1136 (95.2)		17.4 [15.3; 19.8]	
Mixed Italian and other nationality		21 (3.6)	20 (3.3)	41 (3.4)		21.4 [15.3; 25.7]	
Other nationality		10 (1.7)	6 (1.0)	16 (1.3)		-	

N refers to the number of valid responses. If no indicated, the number is 1215. Absolute numbers and percentages. P-value^1 ^refers to the significance of the difference among snackers and no snackers, p-value^2 ^to the difference in BMI across categories of each variable in the snackers only group.

**Table 2:** Description of the children surveyed.

	*N*	*Snacker*	*No snacker*	*Overall*	*p-value^1^*	*BMI*	*p-value^2^*
		*608*	*607*	*1215*		(Snackers only)	
**Gender**					p = ns		p = 0.214
Male		344 (56.6)	331 (54.5)	675 (55.6)		17.8 [15.9; 20.0]	
Female		264 (43.4)	276 (45.5)	540 (44.4)		17.1 [14.9; 20.4]	
**Age (years)**					p < 0.001		p < 0.001
6		128 (21.1)	121 (19.9)	249 (20.5)		16.5 [14.9; 19.0]	
7		120 (19.7)	102 (16.8)	222 (18.3)		18.0 [16.0; 20.0]	
8		167 (27.5)	114 (18.8)	281 (23.1)		15.9 [14.6; 20.7]	
9		102 (16.8)	135 (22.2)	237 (19.5)		17.1 [16.2; 20.4]	
10		91 (15.0)	135 (22.2)	226 (18.6)		17.8 [17.4; 19.8]	
**BMI**	1082	17 [15; 20]	18 [15; 20]	18 [15; 20]	p = 0.120		p < 0.001
Underweight/normal		326 (60.3)	325 (60.1)	651 (60.2)		15.9 [14.6; 16.8]	
At risk of overweight		91 (16.8)	101 (18.7)	192 (17.7)		18.3 [18.1; 20.4]	
Overweight		124 (22.9)	115 (21.3)	239 (22.1)		21.9 [20.7; 23.4]	
**Interest of the child for the snack**	1145				p = ns		p < 0.001
Focused on the product		208 (35.9)	173 (30.6)	381 (33.3)		18.3 [16.5; 20.4]	
Focused on the gadget accompanying the snack		372 (64.1)	392 (69.4)	764 (66.7)		16.6 [14.9; 19.0]	
**Age of the mother at time of child birth**	1187	30 [27; 33]	31 [28; 34]	30 [28; 33]	p < 0.001	17.4 [15.3; 20.0]	
**Time of sleeping of the child per day**	1187				p = 0.002		p < 0.001
At most 8 hours		210 (36.0)	168 (27.8)	378 (31.8)		18.0 [16.0; 20.7]	
More than 8 hours		373 (64.0)	436 (72.2)	809 (68.2)		16.7 [15.2; 19.4]	
**Time when the child wakes up**	1186				p = ns		p < 0.001
7.30 AM or earlier		369 (63.3)	402 (66.7)	771 (65.0)		17.1 [14.9; 19.2]	
Between 7.30 AM and 8.30 AM		101 (17.3)	105 (17.4)	206 (17.4)		18.1 [16.7; 21.0]	
After 8.30 AM		113 (19.4)	96 (15.9)	209 (17.6)		17.1 [16.0; 20.6]	
**Number of meals per day of the child**	1188				p < 0.001		p < 0.001
At most three		69 (11.8)	132 (21.8)	201 (16.9)		16.9 [14.5; 17.9]	
Four		334 (57.3)	334 (55.2)	668 (56.2)		16.8 [15.3; 20.0]	
Five or more		180 (30.9)	139 (23.0)	319 (26.9)		18.3 [16.0; 20.4]	
**Place of meals**	1209				p = 0.088		p = 0.010
At home		538 (89.2)	558 (92.1)	1096 (90.7)		17.6 [15.3; 20.0]	
Outside home		65 (10.8)	48 (7.9)	113 (9.3)		16.5 [14.9; 17.8]	
**The place of meals is changing during the week**	1208				p = ns		p = 0.065
Yes		217 (36.0)	205 (33.9)	422 (34.9)		17.3 [14.9; 20.0]	
No		386 (64.0)	400 (66.1)	786 (65.1)		17.6 [15.4; 20.4]	
**Number of different sport activities per week**	1190				p = ns		p = 0.008
None		175 (29.5)	176 (29.5)	351 (29.5)		17.6 [16.0; 20.4]	
One sport		312 (52.6)	318 (53.3)	630 (52.9)		17.6 [15.2; 20.0]	
Two		82 (13.8)	85 (14.2)	167 (14.0)		17.1 [14.9; 20.4]	
Three or more		24 (4.0)	18 (3.0)	42 (3.5)		16.0 [14.3; 17.5]	
**Time (hours) of sport activities per week**	1190				p = ns		p = 0.133
None		175 (29.5)	176 (29.5)	351 (29.5)		17.6 [16.0; 20.4]	
Between one and three hours		252 (42.5)	246 (41.2)	498 (41.8)		17.8 [14.8; 20.4]	
More than three hours		166 (28.0)	175 (29.3)	341 (28.7)		17.4 [15.3; 19.2]	
**Hours per day watching TV or playing videogames**	1115				p < 0.001		p < 0.001
At most one hour		144 (26.4)	233 (40.9)	377 (33.8)		17.4 [14.5; 18.0]	
Between one and two hours		224 (41.1)	215 (37.7)	439 (39.4)		17.1 [15.3; 20.2]	
More than two hours		177 (32.5)	122 (21.4)	299 (26.8)		17.8 [15.9; 20.4]	
**Hours of studying outside school time**	1041				p = 0.113		p = 0.006
At most three hours		116 (23.2)	131 (24.2)	247 (23.7)		16.8 [14.5; 20.7]	
Between three and seven hours		179 (35.8)	221 (40.9)	400 (38.4)		17.4 [15.3; 19.0]	
More than seven hours		205 (41.0)	189 (34.9)	394 (37.8)		18.1 [16.0; 20.4]	
**What is your perception on the weight of your child**	1204				p = ns		p < 0.001
He/she is underweight		69 (13.9)	60 (11.7)	129 (12.8)		14.5 [13.8; 15.3]	
He/she is normal		428 (86.1)	454 (88.3)	882 (87.2)		16.8 [15.3; 19.0]	
He/she is overweight		101 (16.9)	92 (15.2)	193 (16.0)		20.6 [18.1; 22.8]	
**How much, in Kg**	298	4 [3; 5]	3 [2; 5]	3 [2; 5]	p < 0.001	18.1 [14.6; 21.4]	
**How much (in case of overweight), in Kg**	182	5 [3; 10]	3 [2; 6]	4 [2; 7]	p < 0.001	20.6 [18.1; 22.8]	
**Number of close friends aged 6-10 yrs**	1135				p = 0.188		p = 0.001
None		39 (6.9)	56 (9.8)	95 (8.4)		18.9 [14.3; 22.8]	
Between one and three		100 (17.7)	93 (16.3)	193 (17.0)		17.8 [16.7; 20.8]	
More than three		427 (75.4)	420 (73.8)	847 (74.6)		17.6 [15.3; 19.2]	

N refers to the number of valid responses. If no indicated, the number is 1215. Absolute numbers and percentages. P-value^1 ^refers to the significance of the difference among snackers and no snackers, p-value^2 ^to the difference in BMI across categories of each variable in the snackers only group.

All variables considered were entered into the model "as is", i.e. without any transformation or cutting-off. If a significant non-linearity using a score test was found, in relating the covariate's effect with survival, the specific covariate's effect was modeled using a restricted cubic spline. Selection criteria was the AIC (Akaike Information Criterion) applied backward for selecting significant covariates. The final model for each of the three steps was selected if superior in terms of AIC at a significance level of 0.05 and p-values have been explicitly indicated if below the 0.25 threshold, otherwise the "NS" indication is used.

To account for possible overfitting in the regression models secondary to high ratio between covariates and events, cross-validation and bootstrap (200 runs) techniques were applied. For the logistic regression model, Somer's concordance Index Dxy (the closer to one in absolute value the better) were obtained and evaluated for this purpose.

Multivariate Odds Ratios have been presented along with their 95% confidence intervals for the logistic regression model, whereas for the linear regression model effects on BMI scale have been presented along with their 95% confidence levels.

The statistical significance was settled at a p-value <0.05. The R System (release 2.7.0) statistical package and the Harrell's Design and Hmisc libraries were used for analysis.

## Results

The interviews were balanced by design in terms of geographical distribution, with 326 (26.8%) in the North, 422 (34.7%) in the Center and 467 (38.4%) in the South of Italy.

The sample is described in Table 1 for what concerns family characteristics and in Table 2 for what regards children.

### Snacks

The snacking activity, according to the definition adopted in the study, was eating at least three snacks per day. The distribution of snacks were: Plain cakes 230 (39.2%), Cream filled cakes 336 (57.2%), Chocolate cakes 227 (38.7%), Sugar sodas 299 (50.9%) and other types 16 (2.7%).

### The social network

The network around the reference child is described in Table 3. The social network identified according the strict rules adopted in the study was composed by on average 1.27 friends per child surveyed, slightly but significantly higher for "snackers" than for "no-snackers" (1.40 vs 1.14, p = 0.042).

**Table 3:** Description of the social network of the index child.

	*N*	*Snacker*	*No snacker*	*Overall*	*p-value^1^*	*BMI*	*p-value^2^*
		858	658	1516		(Snackers only)	
**Gender**	1510				p = ns		p = 0.093
Male		468 (54.9)	367 (55.8)	835 (55.3)		17.4 [15.9; 19.0]	
Female		384 (45.1)	291 (44.2)	675 (44.7)		17.6 [16.0; 20.4]	
**Age**	1510				p = 0.005		p < 0.001
At most 8 yrs		498 (58.5)	337 (51.2)	835 (55.3)		16.6 [15.3; 19.2]	
Older than 8 yrs		354 (41.5)	321 (48.8)	675 (44.7)		17.8 [16.6; 20.4]	
**Place of friendship**	1496				p = ns		p = 0.003
School		570 (67.9)	467 (71.2)	1037 (69.3)		17.7 [15.3; 20.4]	
Child of a relative		126 (15.0)	97 (14.8)	223 (14.9)		16.8 [15.8; 20.4]	
Neighbor		114 (13.6)	75 (11.4)	189 (12.6)		17.7 [16.5; 20.7]	
Sport/other places		30 (3.6)	17 (2.6)	47 (3.1)		16.9 [15.9; 17.8]	
**Time (hours) of sport activities per week**	1493				p = 0.002		p < 0.001
None		336 (40.0)	223 (34.2)	559 (37.4)		17.6 [16.5; 20.4]	
Between one and three hours		306 (36.4)	296 (45.3)	602 (40.3)		16.5 [15.0; 20.0]	
More than three hours		198 (23.6)	134 (20.5)	332 (22.2)		17.8 [16.0; 20.4]	
**Number of different sport activities per week**	934				p = ns		p = 0.018
One sport activity		422 (83.7)	362 (84.2)	784 (83.9)		17.1 [15.3; 19.7]	
Two or more sport activities		82 (16.3)	68 (15.8)	150 (16.1)		17.8 [15.9; 20.8]	
**Is the friend eating snacks**	1181				p < 0.001		p = 0.200
Yes		612 (89.5)	379 (76.3)	991 (83.9)		17.8 [16.0; 20.4]	
No		72 (10.5)	118 (23.7)	190 (16.1)		17.8 [16.6; 21.0]	
**Number of snacks per day of the friend**	668				p = ns		p < 0.001
At most one		150 (37.9)	105 (38.6)	255 (38.2)		17.1 [15.8; 18.1]	
Not more than two		120 (30.3)	88 (32.4)	208 (31.1)		18.3 [16.5; 20.4]	
Not more than three		96 (24.2)	53 (19.5)	149 (22.3)		20.4 [17.4; 22.4]	
More than three		30 (7.6)	26 (9.6)	56 (8.4)		16.5 [15.3; 19.8]	
**What is your perception on the weight of your child**	1484				p = 0.82		p < 0.001
He/she is underweight		84 (10.1)	89 (13.7)	173 (11.7)		19.0 [16.7; 20.7]	
He/she is normal		588 (70.5)	432 (66.5)	1020 (68.7)		17.4 [15.4; 19.2]	
He/she is overweight		162 (19.4)	129 (19.8)	291 (19.6)		17.8 [16.5; 20.4]	

N refers to the number of valid responses. If no indicated, the number is 1516. Absolute numbers and percentages. P-value^1 ^refers to the significance of the difference among snackers and no snackers, p-value^2 ^to the difference in BMI across categories of each variable in the snackers only group.

### Multivariable analysis for characterizing the "snacker"

To understand which individual, familiar or social network characteristics are associated with the status of "snacker", a multivariable model has been built, following the variable selection procedure as described in the statistical section among all variables listed in Table 1 and in Table 2. Results of the multivariable model are presented in Table 4.

**Table 4:** Multivariable logistic model for the association with the status of Snacker of selected variables.

	*Effect*	*OR*	*CI*	*95%*
Age of the mother	33 vs. 28	0.79	0.67	0.93
Time (hours) of sport activities per week of the mother	2 vs. 0	0.55	0.38	0.79
Time (hours) of sport activities per week of the child	4 vs. 2	1.36	1.03	1.9
Number of meals/day of the child	5 vs. 2	3.22	1.49	6.98
Number of friends in the network	More than 3 vs. At most 3	1.28	1.01	1.71
Time (hours) of sport activities per week of the friends in the network	4 vs. 2	1.18	1.05	1.43
Is the friend eating snacks?	Yes vs. No	2.44	1.67	3.57

Hours of sport activities of the mother and of the child are showing a non-linear effect (respectively, p = 0.002 and p = 0.0009). Somer's Dxy equal to 0.65.

### Multivariable analysis for the BMI levels in the "snacker" group

To understand which individual, familiar or social network characteristics are associated with any given BMI of the "snacker", a multivariable model has been built, following the variable selection procedure as described in the statistical section among all variables listed in Table 1 and in Table 2 but limited to the group of the 608 "snackers". Results from this multivariable analysis are shown in Table 5.

**Table 5:** Multivariable model for the BMI of the "snacker" child.

Variable	Value	Effect	**S.E**.	*CI*	*95%*
Mother's BMI	4 points increment	0.85	0.12	0.62	1.08
Age (Mother)	5 years increment	0.73	0.17	0.41	1.06
Time studying per day (hours)	6 hrs increment	0.83	0.15	0.53	1.13
Time in sport activities per day (hours)	3 hrs increment	-0.43	0.18	-0.77	-0.08
Time in front of TV per day	≤ 1 h vs. 1-2 hrs	-2.09	0.36	-2.8	-1.38
	>2 hrs vs. 1-2 hrs	-0.26	0.29	-0.83	0.3
Municipality income (low vs. high)		1.82	0.28	1.26	2.37

Linear model for snackers only. The column "value" refers to the interquartile difference for continuous variables or for specific reference categories for categorical variables.

## Discussion

### Familiar and social environment role in determining snacking activity

Results of the limited research on behavioral mediators of familial patterns of overweight indicate that parents' own eating behaviors and parenting practices influence the development of children's eating behaviors, mediating familial patterns of overweight. Parents provide food environments for their children's early experiences with food and eating, and overweight parents seem to select environments that promote overweight among their children [21].

In our study two parental characteristics have been evaluated in relation to child eating behavior: parental BMI and parental habit to practice physical activity.

No association between fathers' BMI and child snacking consumption has been found, while the association between mothers' BMI and child snacking consumption observed in the univariate analysis disappears in multivariable analysis.

On the other hand, in our study children dietary habits seems to be strictly related to mothers' attitude toward physical activity. Indeed, mothers of no-snackers seem to be more prone to healthy behaviors, practicing at least 2 hours of sport activities during the week. But this not necessarily translates in a healthier lifestyle for their children, since no-snackers children tend have a more sedentary lifestyle.

On the contrary, snacking children profile is controversial, since, in according to the literature, they spend more time than no-snackers watching TV, but also, they seem to be more active, since the great part of them practices at least 4 hours per week of physical activity. Differences in family composition could explain this fact: while no-snacking children usually may come from classical families (father, mother and children) and may have not brothers or sisters, snacking children frequently come from numerous, enlarged families. Classical families having one child, may pay more attention on their children lifestyle and on their dietary habits, whereas, in an enlarged familiar environment, parents may have to delegate other people or relatives, such as grandparents, to take care of their children, ending up in a less strict control of their children's dietary habits.

Differently from what described in previous studies on snacking activities [22], in which snacking children are largely described as children usually spending time in solitary activities, in our analysis snacking children, having a more active lifestyle than no-snackers, are part of a more numerous social networks. Thus, instead of observing the well-studied vicious circle in which more isolated children stay at home, spending time in sedentary activity and eating snacks [22], we observed that snacking children are practicing physical activity more than no-snackers and are part of a larger social network. Indeed, in the snackers' social network, most of peers are both practicing sports and eating snacks. The attitude to "regress to the mean" in term of behaviors among peers, with the tendency of mimicking others' behaviors [23] could also contribute to explain these findings.

On the contrary, as a direct consequence of a more sedentary lifestyle, no-snackers children have less social relationships and, similarly to that observed among snackers, their peers tend to share the same eating and leisure time habits.

### Snacking and overweight

The existence of an association between snack consumption and risk of being overweight or obese has been previously evaluated in numerous studies obtaining contrasting results: is was shown in some studies but not in others [9].

In our study we found that the status of snacker seems not to be associated with overweight and/or obesity being the BMI equivalent among snacking and not snacking children. The key factor in explaining such result might be the role of physical activity of the child. In addition to the positive effect of physical activity, by itself, on obesity prevention, also spending leisure time in sportive activities implies being part of a social environment which is definitely a positive one from the point of view of obesity control [24]. Indeed, results do not show an increase of overweight/obesity in relation to snack consumption.

### Factors related to BMI in the snacking group

In the present study, determinants of BMI in snacking group have been explored; according to previous large-scale researches which found correlations between paternal or maternal anthropometric measures and children's adiposity [25-27], also in our analysis an increment in mother BMI seems to contribute to an increment in children BMI. As previously mentioned, this intergenerational relationship reflects genetic, social, cultural, and environmental components since, when parents make food consumption choices (or provide the feasible food consumption set) for children for meals eaten at home, this common environment generates a correlation between generations when both eat unhealthy (or healthy) food together [28].

Moreover, a positive relationship between maternal age and children BMI has also been found, suggesting that younger mothers tend to pay more attention to their children's dietary habits or to encourage them to practice sport.

As expected, energy balance-related behaviors, including sport activity, studying and watching TV are related to children BMI: an increment in the first one induces a BMI reduction, while, more time spent in sedentary activity determines a BMI increasing, as expected.

Finally, as observed elsewhere [29], a positive relationship between low income (indirectly measured here by the municipality income) and body weight seems to exist: the association of child and household food insecurity with childhood overweight has been demonstrated in several studies [30]; moreover, children in low-income families could experience adverse psychosocial conditions which may contribute to poor eating habits and lower physical activity levels [31].

### Study limitations

This study is a survey, without direct interaction between the investigator and the interviewed people and thus relying only on self-reported measurements for what concerns anthropometric characteristics, physical activity levels and dietary patterns. Although this approach has been widely used [32], it still presents the general limitation of such approaches, like reporting bias, barrier effect leading to interview refusal, tendency of ameliorant responses in presence of sensitive questions [31]. The authors used interview and analysis techniques to reduce each of such potential biases, like the involvement of only highly experienced interviewers in interview conduction and multivariable modeling for confounding adjustment.

### Final remarks

No evidence of an association between snacking consumption and overweight status has been shown by our study. This fact can be explained by the different attitude toward physical activity observed in snackers and no-snackers: in fact, differently from common findings in scientific literature, in our study the snacking child can be described as practicing sport more than no-snakers and, as a consequence, having more active peer-to-peer social relationships, mostly related with sport activities. Among snackers, factors traditionally related to body weight such as mother BMI, energy balance-related behaviors and municipality income have been confirmed as major determinants of children BMI.

## Conclusions

Obesity control appeared to be positively influenced by peer-to-peer social relationships, especially those related to sport activities. Snacking behavior didn't present association with overweight and obesity, that instead were linked with families' characteristics, like income or parents' age and BMI. These conclusions point out the chiefly role of energy expenditure in regulating the energy gap, stressing the necessity of to consider obesity as a multifactorial disease, influenced by social behaviors, in first instance related with the familiar environment.

## List of abbreviations

AIC: Akaike Information Criterion; BMI: Body Mass Index; CATI: Computer Assisted Telephone Interview; CDC: Centers for Disease Control and Prevention; CI: Interval of Confidence; NS: Non Significant; OR: Odds Ratio

## Competing interests

The authors declare that they have no competing interests.

## Authors' contributions

DG conceived the study, participated in its design and performed the statistical analysis; FF participated in the design of the study and coordinated and helped to draft the manuscript. MG coordinated data collection and participated in statistical analysis. FZ implemented and supervised survey conduction. SB participated in manuscript drafting. LF participated in manuscript drafting. PB performed the statistical analysis. All authors read and approved the final manuscript.
